# Selective Deletion of the Mechanistic Target of Rapamycin From the Renal Collecting Duct Principal Cell in Mice Down-Regulates the Epithelial Sodium Channel

**DOI:** 10.3389/fphys.2021.787521

**Published:** 2022-01-04

**Authors:** Bruce Chen, Maurice B. Fluitt, Aaron L. Brown, Samantha Scott, Anirudh Gadicherla, Carolyn M. Ecelbarger

**Affiliations:** ^1^Division of Endocrinology and Metabolism, Department of Medicine, Georgetown University, Washington, DC, United States; ^2^Department of Medicine, Howard University, Washington, DC, United States

**Keywords:** kidney, insulin, salt, ubiquitination, sex differences

## Abstract

The mechanistic target of rapamycin (mTOR), a serine-threonine-specific kinase, is a cellular energy sensor, integrating growth factor and nutrient signaling. In the collecting duct (CD) of the kidney, the epithelial sodium channel (ENaC) essential in the determination of final urine Na+ losses, has been demonstrated to be upregulated by mTOR, using cell culture and mTOR inhibition in *ex vivo* preparations. We tested whether CD-principal cell (PC) targeted deletion of mTOR using Cre-lox recombination would affect whole-body sodium homeostasis, blood pressure, and ENaC regulation in mice. Male and female CD-PC mTOR knockout (KO) mice and wild-type (WT) littermates (Cre-negative) were generated using aquaporin-2 (AQP2) promoter to drive Cre-recombinase. Under basal conditions, KO mice showed a reduced (∼30%) natriuretic response to benzamil (ENaC) antagonist, suggesting reduced *in vivo* ENaC activity. WT and KO mice were fed normal sodium (NS, 0.45% Na+) or a very low Na+ (LS, <0.02%) diet for 7-days. Switching from NS to LS resulted in significantly higher urine sodium losses (relative to WT) in the KO with adaptation occurring by day 2. Blood pressures were modestly (∼5–10 mm Hg) but significantly lower in KO mice under both diets. Western blotting showed KO mice had 20–40% reduced protein levels of all three subunits of ENaC under LS or NS diet. Immunohistochemistry (IHC) of kidney showed enhanced apical-vs.-cellular localization of all three subunits with LS, but a reduction in this ratio for γ-ENaC in the KO. Furthermore, the KO kidneys showed increased ubiquitination of α-ENaC and reduced phosphorylation of the serum and glucocorticoid regulated kinase, type 1 [serum glucocorticoid regulated kinase (SGK1)] on serine 422 (mTOR phosphorylation site). Taken together this suggests enhanced degradation as a consequence of reduced mTOR kinase activity and downstream upregulation of ubiquitination may have accounted for the reduction at least in α-ENaC. Overall, our data support a role for mTOR in ENaC activity likely *via* regulation of SGK1, ubiquitination, ENaC channel turnover and apical membrane residency. These data support a role for mTOR in the collecting duct in the maintenance of body sodium homeostasis.

## Introduction

The mechanistic target of rapamycin (mTOR) is a serine-threonine protein kinase involved in the regulation of cell growth, proliferation, metabolism, protein synthesis, and other mechanisms that promote tissue growth (Sarbassov et al., 2004; Betz et al., 2013; Weichhart, 2018). In the renal collecting duct, we and other laboratories have shown that it plays a role in the activation of the epithelial sodium channel (ENaC) (Lu et al., 2010; Pavlov et al., 2013; Lang and Pearce, 2016). Finely tuned regulation of ENaC is essential for electrolyte homeostasis and blood pressure control, as it represents the last regulated site of sodium-(and chloride and potassium) reabsorption along the renal tubule, from glomerulus to the ureter.

There are two distinct, multi-protein complexes that contain mTOR as a component, i.e., mTORC1 and mTORC2. The complexes have marginally different activators and effectors. MTORC1 (inhibitable by rapamycin) integrates input from growth factors, oxidative stress, and nutrients (amino acids). If cellular energy levels appear “high,” mTORC1 activity increases leading to downstream protein transcription and translational events supporting cell and tissue growth (Laplante and Sabatini, 2012a,b). The mTORC1 complex includes mTOR, mammalian lethal with SEC13 protein 8 (MLST8), regulatory-associated protein of mTOR (RPTOR), proline-rich AKT1 substrate, type 40 (AKT1S1/PRAS40), and DEP domain-containing mTOR-interacting protein (DEPTOR), as ancillary proteins (Brown et al., 2018). The second complex, mTORC2 (primarily rapamycin insensitive) appears to be activated solely by growth factors, including insulin and insulin-like-growth factor type 1 (IGF1). MTORC2 contains mTOR, DEPTOR, rapamycin-insensitive companion of mTOR (RICTOR) stress-activated map kinase-interacting protein 1 (mSIN1), proline-rich protein 5 (PRR5), and proline-rich protein-5-like protein (PRR5-L) (Brown et al., 2018) and is involved in regulation of the cytoskeleton *via* stimulation of protein kinase C alpha (PKCα), promotion of cellular survival *via* Akt (protein kinase B) activation, as well as, ion transport and growth *via* serum-and-glucocorticoid-regulated kinase (SGK1) phosphorylation (Sarbassov et al., 2004; Betz et al., 2013; Heikamp et al., 2014).

Prior studies by our group and others revealed that insulin plays a role in sodium reabsorption in the renal collecting duct by activation of the ENaC (Blazer-Yost et al., 1998; Wang et al., 2001; Song et al., 2006; Tiwari et al., 2007; Pavlov et al., 2013). SGK1 is phosphorylated by mTORC2 on serine 422 (an activating phosphorylation in the hydrophobic motif), which partially explains the mechanistic link between insulin and ENaC. Insulin also activates PDPK1 (3-phosphoinositide dependent protein kinase 1), which phosphorylates SGK1 in the activation loop at threonine 256. Activated SGK1 phosphorylates and inhibits NEDD4-2 (an E3 ubiquitin ligase) reducing retrieval of mature channels from the apical membrane. Whether either complex of mTOR has a role in full-differentiation of the collecting duct with its assembly of transporters, channels, and exchangers is not clear.

The consequences of mTOR deletion has been studied in other tissues, including adipocytes and cardiomyocytes (Shan et al., 2016; Baba et al., 2018). Using a strategy similar to our own to delete floxed mTOR, investigators showed knockout (KO) from adipocytes led to a reduction in fat mass and insulin resistance of existing adipocytes (Shan et al., 2016). In another study, deletion of mTOR from cardiomyocytes in an iron-overload mouse model showed mTOR was protective against iron toxicity and apoptosis of the cardiac cells.

Because mTOR in the CD has been shown to regulate ENaC activity in response to insulin, our primary aim was to determine how deletion of mTOR affected ENaC subunit regulation, as well as whole-body electrolyte homeostasis and blood pressure. To accomplish this we bred collecting-duct-principal-cell-targeted mTOR knockout mice utilizing Cre-lox recombination and an aquaporin 2 (AQP2) promoter sequence to target the KO. AQP2 is the most highly expressed transcript in the cortical collecting duct (Lee et al., 2015), and its expression extends from the late distal convoluted tubule (DCT2) through the inner medullary collecting ducts (IMCD). We expected that mTOR deletion might affect activities of both mTORC1 and mTORC2, as it is contained in both complexes. Furthermore, increasing mTORC2 activity has been shown to reduce mTORC1 activity in a feedback loop. Thus, the overall impact on signaling and ENaC function would be difficult to predict without this animal model.

## Materials and Methods

### Generation of Collecting-Duct-Principal-Cell Targeted Mechanistic Target of Rapamycin Knockout Mice

Mechanistic Target of Rapamycin knockout targeted to the kidney collecting duct principal cells (*Aqp2*^*cre*^; *Insr*^*flox/flox*^) were generated at Georgetown University on C57Bl6 background. Aqp2^*cre*^ transgenic mice originating from the colony of Don Kohan (University of Utah) (Stricklett et al., 1999) and homozygously floxed *mTOR* mice (B6.129S4-Mtor^*tm1.2Koz*^/J, JAX Laboratories) were crossed for two generations to produce *Aqp2*^*cre*^ positive; *mTOR*^*flox/flox*^ (“KO”) and *Aqp2*^*cre*^ negative, homozygously floxed littermates (“WT”). Mice were genotyped for the presence of Aqp2^*cre*^ by standard PCR on tail snips. The mice were cared for under the guidelines and approved IACUC protocols of the U.S. National Institutes of Health (NIH) Guide for the Care and Use of Laboratory Animals.

### Low- Na+ (LS) Diet Study Design, Blood Pressure, and Urine and Plasma Analyses

The ability of the mice to adapt to a 1-week feeding of a very-low Na^+^ (LS, 0.01–0.02% Na+, Envigo TD.09228) was tested on adult (4–8 months of age) male and female KO mice. Mice were fed this diet or a normal salt (NS) diet (1% NaCl, Purina 5001), *ad libitum* with free-access to drinking water (*n* = 6/genotype/treatment/sex). In some sets of mice, urine was collected (24 h) in mouse metabolic cages (MMC100, Hatteras Instruments), while mice had free access to diet and water. In another set of mice, blood pressure was measured by tail-cuff plethysmography (Coda^®^ High Throughput System, Kent Scientific Corporation). Mice were euthanized by Inactin (Thiobutabarbital, Sigma) overdose and blood was collected from the heart and analyzed with a VetScan^®^ iSTAT1 chemical analyzer and EG6 cartridges (iSTAT, Abbott). After perfusion with phosphate-buffered saline (PBS), kidneys were rapidly removed and weighed. Plasma and urine aldosterone was measured by an ELISA (Cayman Chemical). Urine electrolytes were measured by a Medica EasyLyte Analyzer, and/or by flame photometry (BWB-XP flame photometer, BWB Technologies).

### Benzamil-Sensitivity

The natriuretic response to benzamil (ENaC antagonist) was used to gauge ENaC activity in the mice. In an acute test, mice (on NS) were administered benzamil chloride (Sigma) intraperitoneally, dissolved in 0.45% NaCl in sterile water (0.2 ml/30 g⋅bw) at about 11:00 a.m. Urine was collected for 4 h in mouse metabolic cages with water and no food. Urine Na+ concentration (EasyLyte Electrolyte Analyzer, Medica) and volume were measured.

### Cre-Reporter Staining

To test localization of Cre-recombinase activity, AQP2-Cre transgenic female mice were crossed with male reporter mice (The Jackson Laboratory, Bar Harbor, ME; catalog no. 002073 B6;129-Gtrosa26tm1Sor, Soriano Line) (Friedrich and Soriano, 1991). These mice were homozygous for a transgene with loxP sites flanking a DNA stop sequence proceeding a LacZ gene. Recombination without the stop sequence allowed for expression of β-galactosidase, which produced a blue precipitate with use of the β-Gal Staining Kit (Invitrogen, K146501).

### Immunohistochemistry

The perfused left kidney was prepared for immunohistochemistry (IHC) by coronal bisection followed by immersion fixation in 4% paraformaldehyde overnight. This was followed by a buffer exchange to 30% sucrose in PBS for longer-term storage prior to embedding into paraffin (Histopathology & Tissue Shared Resource, Georgetown University). Sections (5 μm) were prepared for IHC. Immunoperoxidase-based staining for mTOR, AQP2, α-, β-, and γ-ENaC was performed as previously described (Tiwari et al., 2007). Double-staining for AQP2 (gray) and mTOR (brown) was accomplished by first localizing mTOR (mTOR polyclonal rabbit, PA5-34663 Invitrogen) followed by a 30-min incubation with biotinylated horse anti-rabbit IgG antibody (H + L) secondary (BP-100-50, Vector Laboratories) and DAB (3,3′-Diaminobenzidine)/hydrogen peroxide brown precipitation. The mTOR-primary/secondary antibody complex was stripped off @ 50^°^C for 1 h using glycine/20% SDS at pH 2.0. This was followed by washing and reprobing with AQP2 primary (our own polyclonal rabbit), secondary (as above) and alkaline phosphatase black staining with ImmPACT^®^ (Vector Black, Vector Laboratories, Burlingame CA) which produced a gray precipitate. For ENaC subunits, the ratio of stain density near the apical membrane (∼0–10% of the distance from apical to basolateral membrane) to signal within the remaining 90% of this distance was determined These ratios were calculated for 10 selected cells from each mouse stained section and a median ratio determined for each mouse for each subunit (Image J, NIH). Stained sections were analyzed in a genotype- and treatment-blinded fashion.

### Western Blotting

The right kidney was dissected into cortex, inner medulla, and inner stripe of the outer medulla and homogenates were prepared from each, as we have previously described (Ecelbarger et al., 2000). Western blotting was conducted as previously described (Tiwari et al., 2006). MTOR polyclonal antibody was obtained from Invitrogen (PA5-34663). We used our own rabbit polyclonal antibodies against aquaporin-2 (AQP2), aquaporin-3 (AQP3), β-ENaC (against AA 617-638), γ-ENaC (AA 629-650), and NaPi-2 (AA 614-637). These antibodies were made against sequences previously described by Knepper and associates (Nielsen et al., 1993; Ecelbarger et al., 1995; Masilamani et al., 1999; Kim et al., 2000). Rabbit polyclonal antibodies against UT-A1 (403) and α-ENaC (AA 46-68, L909) were kind gifts from the laboratory of Mark A. Knepper (Epithelial Systems Biology Laboratory, NHLBI, NIH). The rabbit polyclonal Rhbg antibody was a kind gift from the laboratory of David Weiner (University of Florida). The rabbit polyclonal NHE3 antibody was from StressMarq Biosciences (catalog #SPC-400). The rabbit polyclonal NBCe1 antibody was from Proteintech (catalog #11885-I-AP). The rabbit oligoclonal SGK1 antibody was from Invitrogen (22 HCLC). The rabbit polyclonal p-^*S*422^SGK1 antibody was from Santa Cruz (catalog #16745R). Membranes were exposed to chemiluminescence substrate (Pierce) for 1–5 min. The images were then obtained using film processed in a darkroom or by a digital imager (Amersham 600). Membranes were stained with 0.1% Ponceau-S stain (Thermo Fisher Scientific) directly after blotting to address loading, protein transfer, and allow for specific band normalization. Occasionally, the lower portion of the blot was probed with β-actin for normalization.

### Ubiquitination of Epithelial Sodium Channel

To determine whether KO or LS diet affected ubiquitination of ENaC subunits, we used the UbiQuant S quantitative Ub-substrate ELISA (LifeSensors, Inc.) kit. All ubiquitinated proteins were immunoprecipitated into the plate wells containing anti-ubiquitin antibody. Then the wells were probed with either α-, β-, and γ-ENaC polyclonal antibodies. After washes, secondary antibody was applied, followed by more washing then addition of chemiluminescence substrate. Chemiluminescent signal was recorded using an Amersham 600 Imager. Signal intensity of the well is related to the degree of ubiquitinated ENaC subunit.

### Statistics

Data are presented using mean ± standard error means (SEM). Unpaired t-test was used when comparing two groups. With regard to urine data, mixed effected model (REML) or two-way (Time × Genotype) were used. Two-way ANOVA followed by Tukey’s multiple comparisons testing was used to analyze western blotting data and membrane-to-cytosol density ratios for ENaC subunits. *P* < 0.05 was considered significant and corrected for multiple comparisons (GraphPad Prism 8.1.2).

## Results

### Characterization of the Principal-Cell-Select Mechanistic Target of Rapamycin Knockout

**FIGURE 1:**
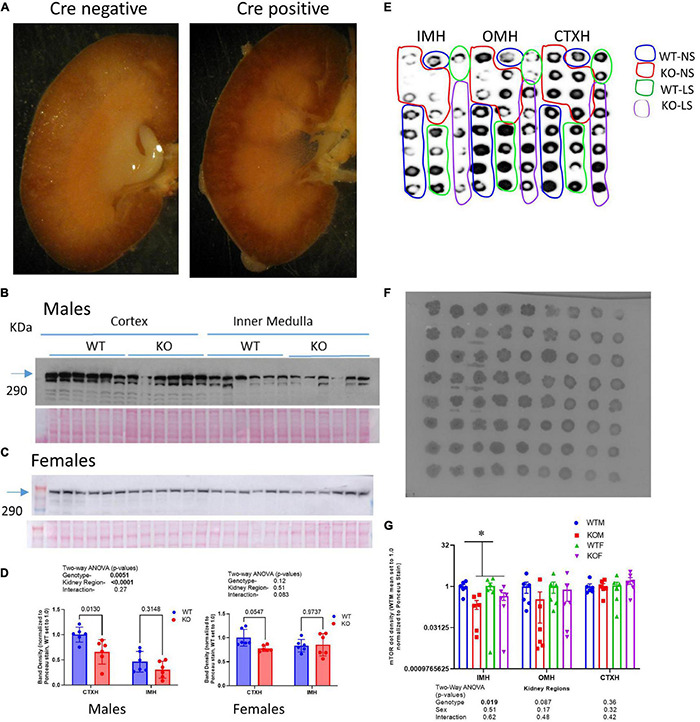
Characterizing the CD-principal-cell targeted mTOR deletion. **(A)** To evaluate cellular location of Cre-recombinase activity, LacZ reporter mice were crossed with Aqp2^*cre*^ transgenic mice and the kidneys of offspring probed for β-galactosidase activity- blue stain; left (Cre-negative); right (Cre-positive) demonstrating staining specific to renal collecting duct in Cre positive mice. mTOR CD-KO mice were generating by crossing male Mtor^*tm1.2Koz*^/J with female Aqp2^*cre*^ mice. Western blots of cortex and inner medulla homogenates (equal amounts of protein were loaded in each lane, 10 μg/lane) from wild-type (WT) and knockout (KO) **(B)** male and **(C)** female mice (*n* = 6/group) probed with anti-mTOR polyclonal antibody; **(D)** densitometric summaries of western blotting of mTOR in cortex (CTXH) and inner medullary homogenates (IMH, mean ± sem); **(E)** dot blot of inner medulla (IMH), outer medulla (OMH), and cortex (CTXH) homogenates (2 μl/dot) from WT and KO male and female mice (*n* = 6/group) probed with anti-mTOR antibody. **(F)** Ponceau S staining of proteins on dot blot for loading correction of mTOR dot blot; **(G)** density summary of dots normalized by Ponceau intensity. Two-way ANOVA (genotype × sex) is shown below bars; * indicates a significant (*p* < 0.05) difference between WT and KO.

Aquaporin-2-promoter-driven Cre recombinase localization was assessed by crossing AQP2-Cre transgenic mice with a LacZ reporter strain. In offspring that harbored the AQP2-Cre transgene, as predicted, β-galactosidase precipitate was localized to collecting duct principal cells, and highly concentrated in the inner medulla (Figure 1A). Demonstration of the mTOR protein deletion from principal cells was assessed by IHC, western and dot blotting. Using western blotting of whole-cell homogenates of cortex and inner medulla from males (Figures 1B,D left panel) and females (Figures 1C,D right panel), we found in general, reduced mTOR protein band densities in the KO cortex relative to WT. In contrast, band densities in the inner medullary homogenates were more variable and not significantly different between genotypes. Moreover, in males, mTOR showed greater density in cortex than in inner medulla, but in females, expression was similar between the regions. As the CD principal cell is a minority cell type in tissue homogenates, making the deletion difficult to observe by traditional western blotting, we also assessed the KO by dot blotting. We evaluated cortex, inner stripe of the outer medulla, and inner medulla homogenates from both sexes and genotypes on a single blot. Here we found (Figures 1E–G), a significant reduction (*p* = 0.019) in mTOR dot density in the inner medullary homogenates in the KO mice. Furthermore, there was a trend for a reduction in dot density in the KO in the outer medullary homogenates (OMH), although this did not reach significance (*p* = 0.087). No differences were observed using dot blotting in the cortex homogenates (CTXH). Finally, using the dot blotting approach for mTOR, we did not observe any sex differences.

### Mechanistic Target of Rapamycin Immunohistochemistry in Renal Tubules

**FIGURE 2:**
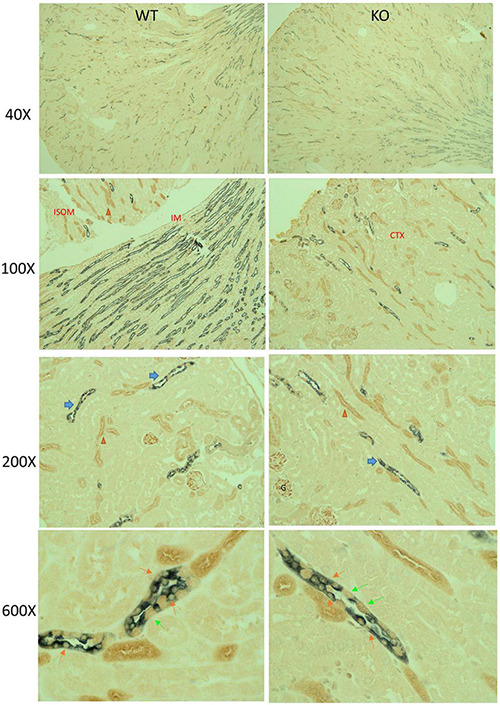
Mechanistic Target of Rapamycin (mTOR) immunohistochemistry (IHC) in mouse kidney. Paraffin-embed kidney coronal sections were prepared for double-labeling IHC by standard chromogenic approaches; mTOR (brown) and AQP2 (gray) in male KO and WT mouse kidneys under control dietary conditions. Sections were imaged at 40×, 100×, 200×, and 600× total magnification, as indicated. At 200×, blue and orange arrows point to collecting duct and thick ascending limbs, respectively, which displayed relatively stronger mTOR labeling than other cells of the kidney (including proximal tubule). Glomerular (G) also displayed strong mTOR labeling. Under 600×, orange and lime-green arrows indicate principal and intercalated cells, respectively. Principal cells were identified by dual gray and brown label. KO mice had reduced brown labeling in cells co-staining with gray.

Immuno-based IHC for mTOR (brown) and AQP2 (gray) in male KO and WT mouse kidneys under control conditions is shown in Figure 2. In general, mTOR labeling was strongest in the thick ascending limb, distal tubule, and collecting duct (CD), relative to proximal tubule. Glomeruli also labeled with mTOR antibody. Dual labeling with AQP2 was used to identify CD principal cells. In the CD, qualitatively we observed less brown staining in cells co-stained with gray in the KO mice (orange arrows). We also observed strong labeling of mTOR in the intercalated cells in both genotypes (green arrows). The CD appeared, in general, of normal diameter in the KO, with no obvious differences in the ratio of principal to intercalated cells.

### Urinary Electrolyte Excretion With a Low-Na+ Diet

**FIGURE 3:**
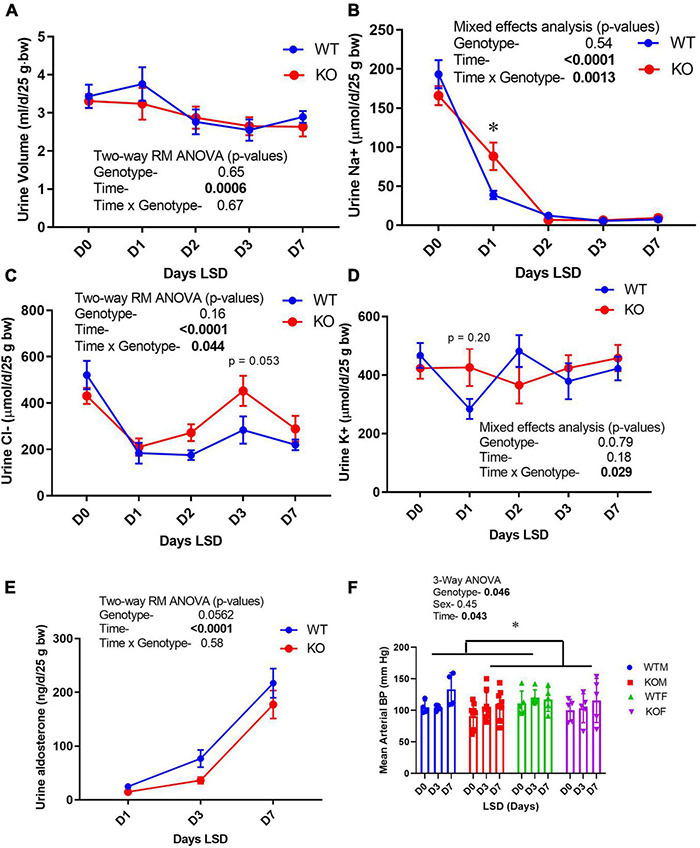
Urine electrolyte excretion, blood pressure, and urine aldosterone under normal and low- Na+ diet. Urine (24 h) was collected on male mice in metabolic cages first under normal diet (D0) and then under 7 days of low- Na+ diet (LSD). Blood pressure (in both sexes) was recorded on a separate group of mice in the baseline and after 3 and 7 days of LSD. Mice were taken out of cages to reduce stress on days 5–6. Volume was recorded and electrolyte concentrations and aldosterone determined on urine and expressed as total excreted per day (d) normalized to 25 g body weight. **(A)** urine volume; **(B)** urine Na+ (volume × concentration); **(C)** Cl−; **(D)** K+, **(E)** aldosterone excretion patterns; **(F)** mean arterial blood pressure (tail cuff) in WT (blue) and KO (red) mice (*n* = 6 mice/genotype); results of two-way ANOVA provided in figure inset. KO mice showed attenuation in the reduction in urine Na+ on Day 1, and modestly but significantly reduced blood pressure. *indicates a significant difference due to genotype (*p* < 0.05) as determined by 2-way ANOVA.

Because the CD is essential in the fine-tuning of Na+, Cl−, and K+ excretion, electrolyte excretion patterns upon switching from a normal (0.45%) to a low- (<0.02%) Na+ diet were evaluated (Figure 3). In the males, both genotypes experienced a slight decline in urine volume with increasing days on LS diet, with no differences between groups (Figure 3A). Daily excreted amounts of electrolytes, i.e., Na+ (Figure 3B), Cl− (Figure 3C), and K+ (Figure 3D) did not differ between genotypes on D0, indicating that WT and KO mice ate approximately the same amount of chow, and were in balance before initiating LS feeding. D1 Na+ losses were significantly higher in the KO, indicating retarded ability to rapidly reduce Na+ excretion. By day 2, however, Na+ losses were not different between genotypes. The significant (time × genotype) interaction, demonstrated that the WT and KO did not respond the same over time. Urine Cl− excretion also showed the time × genotype interaction, in that the WT were able to reduce urine Cl− excretion to a greater extent on the LS diet, but this difference was delayed and sustained in the KO (relative to Na+ response, Figure 3C). Urine K+ also showed a time × genotype significant interaction in that there was a tendency for urine K+ to be higher on D1 and lower on D2 in the KO, in relation to WT (Figure 3D). Taken together these findings suggest a “sluggish,” but eventually full capacity, adaptive response to the LS diet in the KO. Urine aldosterone was also measured (Figure 3E) and found to be increased as expected by LS diet in both genotypes. Surprisingly, the KO had a trend for suppressed urine aldosterone (*p* = 0.052). Blood pressures (Figure 3F) were measured in both male and female mice and found to be slightly, yet significantly, lower in the KO mice overall (*p* = 0.046). LS diet did not reduce blood pressure. Urine volume, Na+ and K+ measures are shown in Supplementary Figure 1 in female mice. Female KO mice had an elevated basal excretion rate of urine Na+ with no genotype differences on subsequent daily collections. No differences in urine Cl− or K+ were observed in the female mice.

### Reduced Epithelial Sodium Channel Activity and Protein Abundance in Knockout Mice

**FIGURE 4:**
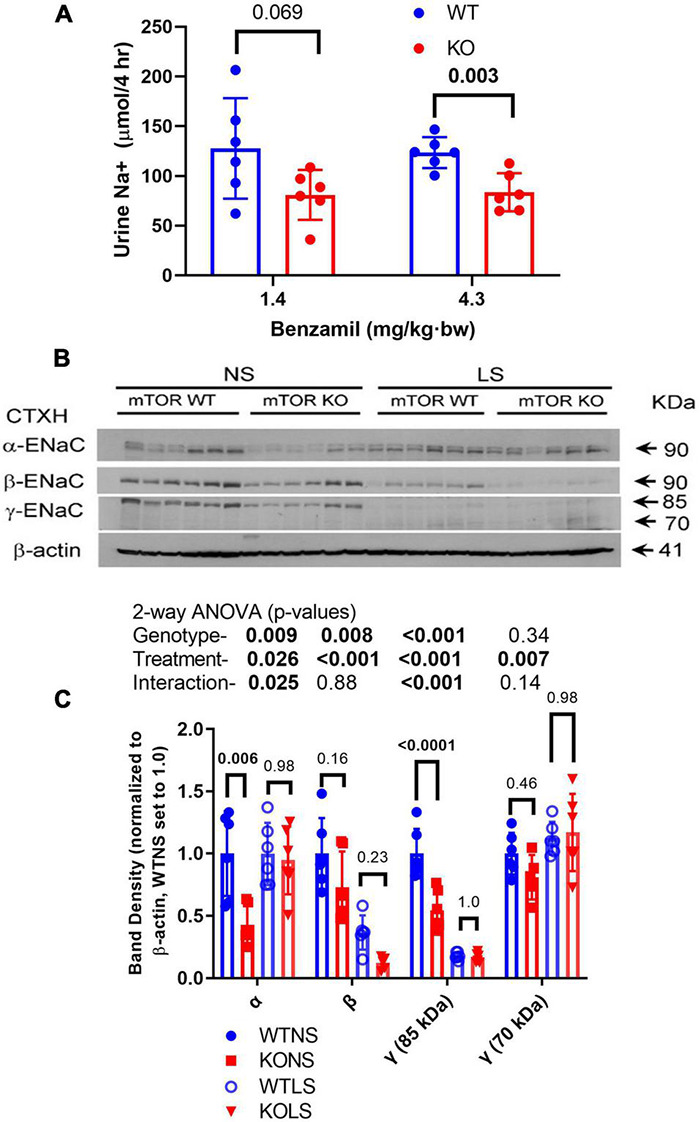
Epithelial sodium channel (ENaC) activity and expression in kidney is reduced in KO mice. **(A)** Natriuretic sensitivity to the ENaC-select antagonist, benzamil was used as a surrogate for *in vivo* ENaC activity. Male mice (*n* = 6/genotype) were given a single intraperitoneal injection of benzamil @ 1.4 mg/kg⋅bw, urine collected in the subsequent 4-h period then measured for volume and Na+. The experiment was repeated the following week with 4.3 mg/kg⋅bw dose. **(B)** Male mice were fed NS or LS diet for 7-days (*n* = 6/genotype/diet) then euthanized and kidneys harvested. **(B)** Western blots of kidney cortex homogenates loaded with 30 μg/lane were probed with rabbit polyclonal antibodies against α-, β-, or γ-ENaC as indicated. A reprobe with β-actin (representative image) was used to normalize bands. **(C)** Band density summary (mean ± sem) and results of 2-way ANOVA (genotype × treatment). The major bands for all three subunits were significantly reduced (*p* < 0.05) in the KO, relative to WT. The LS diet also significantly affected abundance of all bands analyzed.

Because ENaC is a major regulator of sodium reabsorptive activity in the CD, we gauged *in vivo* activity of ENaC by benzamil (BNZ, ENaC antagonist) sensitivity (Figure 4A). WT and KO mice (male) were treated intraperitoneally with BNZ at two different doses and acute natriuretic response was measured. KO mice demonstrated relatively reduced (40–50%) urine Na+ excretion (in the 4-h post-injection collection) suggesting reduced ENaC activity. Kidneys were harvested from mice after 7-days of a normal or a low-NaCl diet. Western blotting of kidney cortex homogenates for the male mice are shown in Figure 4B with a summary of the density data provided in Figure 4C (females in Supplementary Figure 2). In the males, the major bands for all three subunits were significantly reduced in the KO relative to WT (two-way ANOVA). The LS diet attenuated differences between the genotypes and showed the expected increases in the 70-kDa band (cleavage band) for γ-ENaC in both genotypes. As has been reported previously (Masilamani et al., 2002), the major bands for β- and γ-ENaC were reduced by dietary Na+ restriction. In the female mice, a similar, but not identical pattern of ENaC-subunit-abundance regulation was observed. α-ENaC densities were significantly lower (∼40%) in the KO females under both diet conditions; however, no genotype difference was observed for β-ENaC, and γ-ENaC had a strong significant interaction in that it was reduced (relative to WT) with NS diet, but increased with LS diet. The LS diet produced similar responses in the females, as in the males, i.e., increased 70-kDa band for γ-ENaC and a reduction in the density of the β-ENaC band.

### Reduced Apical Localization of the γ-Subunit of ENaC in Cortical Collecting Duct of Knockout Mice

**FIGURE 5:**
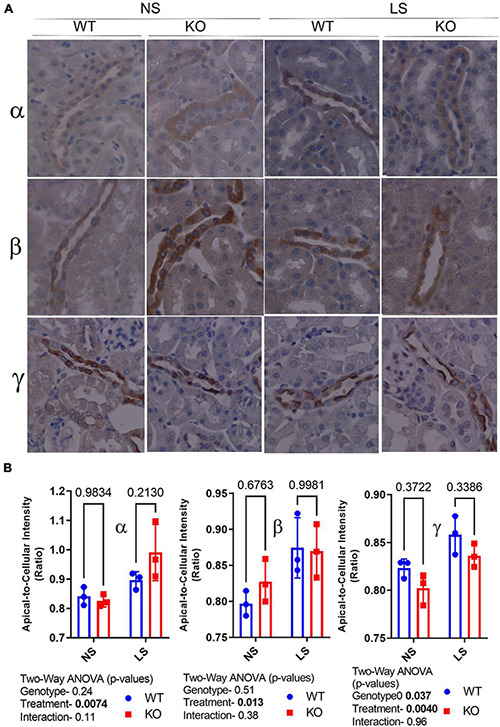
Epithelial sodium channel (ENaC) subunit principal cell localization. **(A)** Immunoperoxidase-staining was used to assess intensity and sub-cellular localization of α- (top panel), β- (middle panel), and γ-(bottom panel) ENaC in 7-day NS or LS-treated male WT and KO mice. **(B)** Summary analysis of mean pixel intensity (Image J, NIH) in the apical-to-cellular regions of the principal cells (*n* = 10 replicate cells/stained section, *n* = 3 mice/group). LS diet increased relative apical localization of all ENaC subunits Moreover, γ-ENaC apical staining was lower in the KO (relative to WT).

Epithelial sodium channel activity is regulated by channel number, open probability, and subcellular localization. Thus, we evaluated subcellular distribution of the ENaC subunits by IHC (Figure 5). Representative labeling of a cortical CD in a male mouse in each of the treatments/genotypes is shown in Figure 5A as indicated. Figure 5B shows apical-to-cellular intensity of signal. Two-way ANOVA showed LS diet led to increased relative apical localization of α-, β-, and γ-ENaC staining. Moreover, γ-ENaC apical staining was lower in the KO (relative to WT).

### Evidence of Increased α-Epithelial Sodium Channel Ubiquitination in Knockout Mice Cortex Homogenates

**FIGURE 6:**
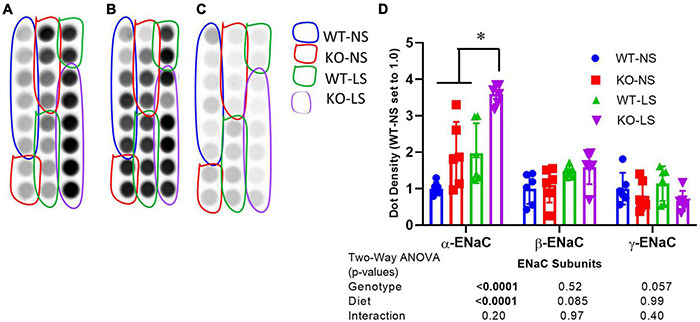
Ubiquitination of α-ENaC increased in KO kidney. Ubiquitination of **(A)** α-, **(B)** β-, and **(C)** γ-ENaC in kidney cortex homogenates of LS and NS-treated male and female mice was assessed by an ELISA-based approach first immunoprecipitating all ubiquitinated proteins and then probing with anti-ENaC subunit antibodies. **(D)** Density summary of dots (mean ± sem) normalized to WT-NS (*n* = 6/genotype/sex/treatment). α-ENaC showed increased ubiquitination in the KO and reduced ubiquitination with LS diet. *indicates a significant difference (*p* < 0.05) by multiple comparisons testing following a significant 2-way ANOVA.

To determine if the reduction in ENaC subunit protein in the KO may be due to enhanced protein degradation, we assessed ubiquitination of ENaC subunits in male mice fed normal or LSD (Figure 6). All ubiquitinated proteins (in cortex homogenate) were immunoprecipitated onto plates which were subsequently probed with ENaC antibodies. We found α-, but not β- or γ-ENaC had significantly increased ubiquitination (although γ- showed a strong trend, *p* = 0.057 in this direction). Furthermore, samples from the LS mice also showed higher levels of α-ENaC ubiquitination, which might represent elevated turnover (both production and degradation).

### Reduced Aquaporin-2 and Rhbg in Kidney of Knockout Mice

**FIGURE 7:**
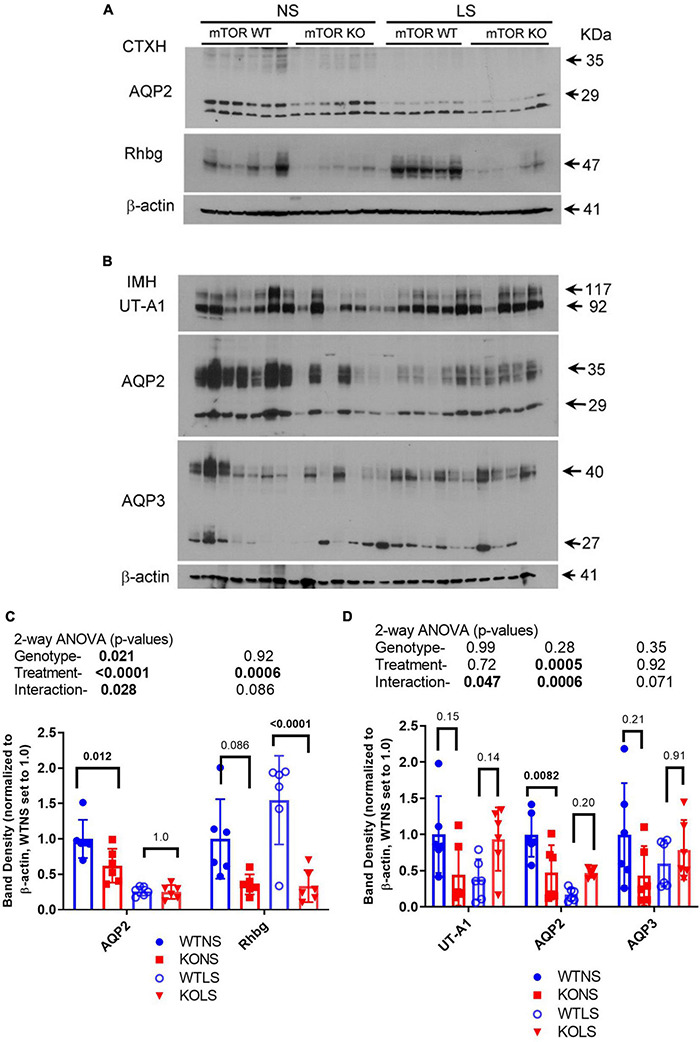
Protein abundances of selected transport/channel proteins in collecting ducts. Western blots of kidney **(A)** cortex homogenates or **(B)** inner medullary homogenates from NS and LS diet fed male WT and KO mice were probed with antibodies against candidate proteins. **(C,D)** Band density (mean ± sem) summary (*n* = 6 mice/treatment/genotype) and two-way ANOVA *p*-values for major bands. AQP2, aquaporin-2; AQP3, aquaporin-3; Rhbg, ammonium transporter; UT-A1, urea transporter. KO mice had reduced protein levels for AQP2 and Rhbg in cortex. Inner medullary protein levels were less affected by the KO.

We next assessed the protein abundance of other major transport/channel proteins in the CD in the male mice to determine whether they were similarly down-regulated in the KO (Figure 7). Aquaporin 2, another apical channel of the principal cells (DiGiovanni et al., 1994), was (like ENaC) significantly reduced in the KO, in cortex (Figures 7A,C) and medulla (Figures 7B,D), with NS, but not LS diet. LS diet also markedly reduced AQP2 abundance in both kidney regions. The urea transporter (UT-A1) of collecting duct principal cells showed a significant interactive term between treatment and genotype (Figures 7B,D) in that it was reduced in the KO under NS, but increased under LS diet. Rhbg, a collecting duct ammonium transporter found on the basolateral side of both intercalated and principal cells (Verlander et al., 2003) was down-regulated in the KO. AQP3, a basolateral water channel in principal cells (Ecelbarger et al., 1995), was not significantly different between genotypes or due to treatment.

### Regulation of Non-collecting Duct Major Sodium Transporters and Exchangers

**FIGURE 8:**
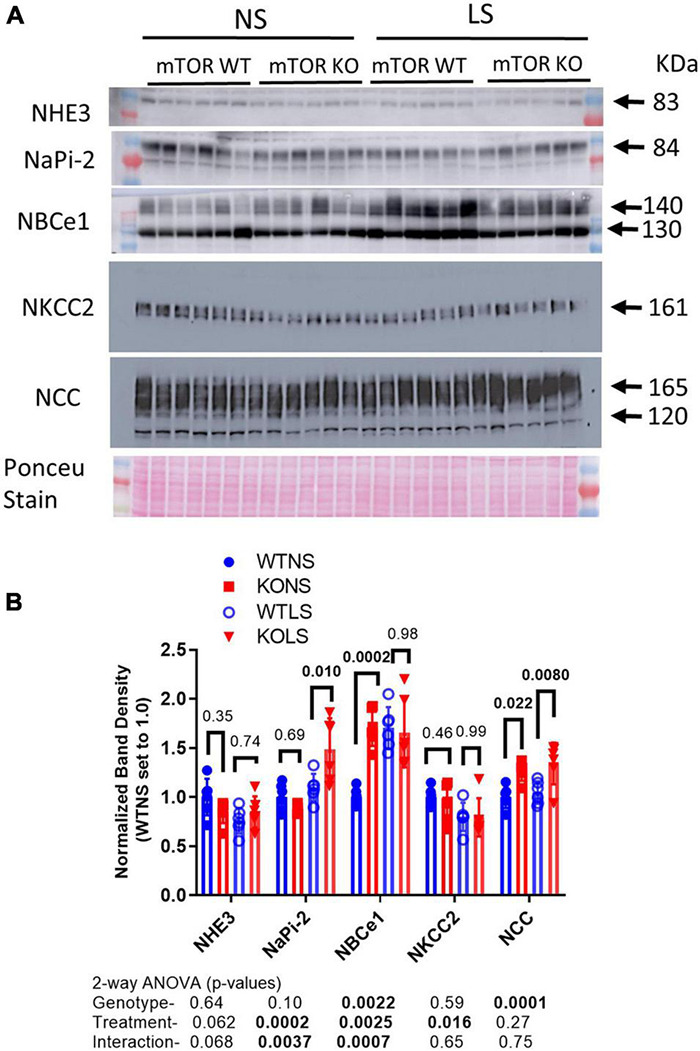
Abundances of non-collecting-duct sodium reabsorptive proteins. Western blots of kidney **(A)** cortex homogenates from NS and LS diet fed male WT and KO mice were probed with antibodies against candidate proteins. **(B)** Band density (mean ± sem) summary (*n* = 6 mice/treatment/genotype) and two-way ANOVA p-values for major bands. NHE3, sodium hydrogen exchanger, type 3; NaPi-2, sodium phosphate cotransporter, type 2; NBCe1, electrogenic sodium bicarbonate cotransporter, type 1; NKCC2, bumetanide-sensitive sodium potassium 2 chloride cotransporter, type 2; NCC, thiazide-sensitive sodium chloride cotransporter. KO mice had increased protein levels of NaPi-2 under LS diet, NBCe1 under NS diet, and NCC under both diets relative to WT.

To determine if sodium transport might be upregulated at a different site along the renal tubule to compensate for reduced ENaC expression, we evaluated major sodium transporters in proximal tubule, thick ascending limb, and distal convoluted tubule (Figure 8). There was no effect of diet or genotype on the expression of the sodium hydrogen exchanger, type 3 (NHE3 or *slc9a3*). There was a significant effect of treatment (LS diet), as well as, a significant interaction between treatment and genotype for the sodium phosphate cotransporter, type 2 (NaPi-2, *slc34a2*). NaPi-2 was increased by LS diet in the KO but not the WT. Similarly, there was a significant interactive term for the sodium bicarbonate cotransporter (NBCe1, *slc4a4*) in that it was increased in the KO under normal, but not LS diet. The bumetanide-sensitive sodium potassium 2 chloride cotransporter, type 2 (NKCC2, *slc12a1*) was slightly, but significantly reduced by LS diet, but not different between genotypes, and the thiazide-sensitive sodium chloride cotransporter (NCC, *slc12a3*) was increased in the KO under both dietary conditions.

### Blood Na+ Is Reduced in Knockout Mice

**FIGURE 9:**
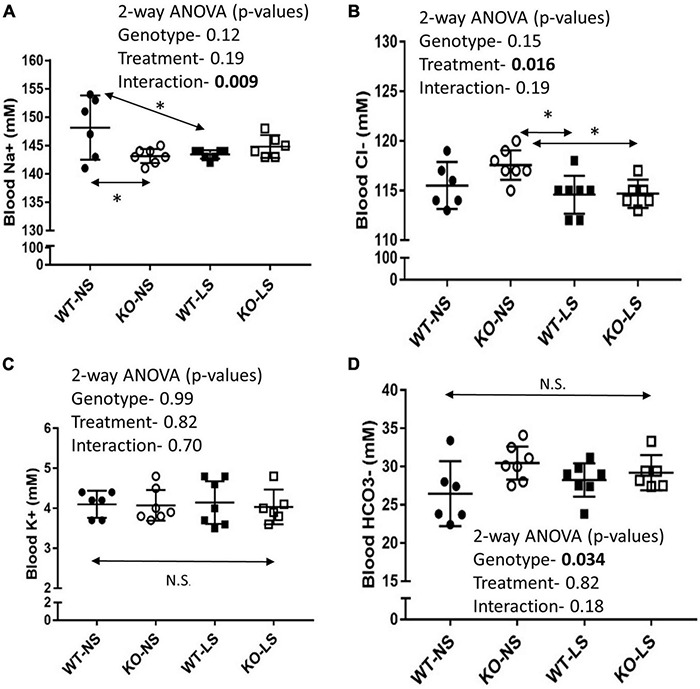
Blood chemistry under normal and low-Na+ diet- Blood chemistry was performed by iSTAT after 7-days of NS or LS diet. **(A)** Na+, **(B)** Cl−, **(C)** K+, **(D)** HCO_3_− (*n* = 6 mice/group). Two-way ANOVA *p*-values are shown within the panels. KO mice has reduced blood Na+ under NS, but not LS diet. LS reduced blood Cl− in both genotypes. No effects on blood K+ were noted. KO mice had significantly higher blood HCO_3_− under both diets. * indicates a significant (*p* < 0.05) difference between groups.

Mean Na^+^ concentrations in the blood were significantly lower in the KO mice under NS diet (Figure 9A). LS diet reduced Na^+^ concentrations in the blood of the WT, so they were no longer different from KO. Blood Cl− concentrations fell significantly in KO on LS diet, but not in WT (Figure 9B). No differences were observed for blood K+ (Figure 9C). Blood bicarbonate levels were significantly higher in the KO under both diets (Figure 9D). Additional whole blood measures (by iSTAT analysis) and plasma (ELISA) are provided in Table 1. LS diet increased blood glucose, hemoglobin, and aldosterone concentrations in both genotypes. Blood chemistry conducted in the female mice are provided in Supplementary Table 1. No genotype differences in blood pH were found in the female mice; however, the KO females had significantly higher blood K+, hematocrit, and hemoglobin, and bicarbonate trended higher (*p* = 0.11), as compared to WT.

**TABLE 1:** Weight and Blood Parameters in male CD-PC mTOR KO mice under normal and low-NaCl diets.*

	WT-NS	KO-NS	WT-LS	KO-LS	Two-Way ANOVA (*p*-values)		
					Genotype	Diet	Interaction
Final body weight (g)	32.9 ± 2.0	27.1 ± 1.5	26.7 ± 1.7	28.2 ± 1.0	0.20	0.13	0.033
Kidney weight (g/25 g⋅bw)	0.125 ± 0.006	0.132 ± 0.002	0.138 ± 0.002	0.135 ± 0.002	0.46	0.026	0.18
BUN (mg/dL)	26.5 ± 0.8	25.6 ± 0.6	26.0 ± 0.8	24.7 ± 1.1	0.20	0.42	0.81
Glucose (mg/dL)^†^	194 ± 8^AB^	185 ± 8^B^	205 ± 5^AB^	212 ± 2^A^	0.94	0.005	0.24
Hct (%PCV)	33.7 ± 0.8	32.1 ± 0.8	33.7 ± 2.0	35 ± 0.7	0.93	0.26	0.28
Hb (g/dL)	11.4 ± 0.3	10.9 ± 0.3	11.9 ± 0.4	11.9 ± 0.2	0.38	0.026	0.43
pH (log H+)	7.18 ± 0.02^B^	7.31 ± 0.03^A^	7.23 ± 0.01^AB^	7.26 ± 0.02^AB^	0.001	0.94	0.053
Beecf (mM)	−1.83 ± 1.96^B^	4.28 ± 0.99^A^	0.71 ± 1.06^AB^	2.33 ± 1.31^AB^	0.009	0.83	0.107
AnGap (mM)	10.3 ± 4.4	−0.9 ± 1.3	4.8 ± 0.7	3.8 ± 1.8	0.016	0.87	0.04
Aldosterone (pM)	94 ± 17	60 ± 11	191 ± 70	317 ± 136	0.54	0.026	0.29

*Mean ± sem (n = 6, 7, 7, and 6 for WT-NS, KO-NS, WT-LS, KO-LS, respectively).

*^†^Non-fasted; Superscript letters indicate significant differences between groups as determined by Tukey’s multiple comparisons test; “A” (assigned to highest mean) is significantly different from “B,” but not “AB.”*

### Reduced Phosphorylation of Serum Glucocorticoid Regulated Kinase on Serine 422 in Knockout Mouse Kidney

**FIGURE 10:**
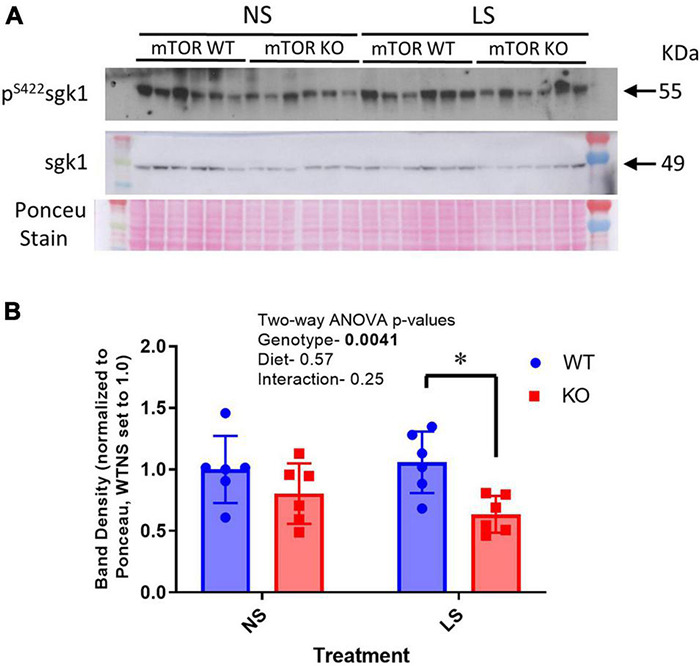
Phosphorylation of SGK1 is reduced in KO kidney. **(A)** A western blot of cortex homogenates from NS and LS diet fed male mice probed with an antibody against phosphorylated SGK1 (pS422). **(B)** Mean band densities (mean ± sem) normalized to Ponceau stain. Two-way ANOVA *p*-values are shown within the panel; * indicates a significant (*p* < 0.05) difference between groups.

We predicted the reduction in sodium retentive-ability with LS diet in the KO mice and increased ubiquitination of ENaC might have been due to a reduction in phosphorylation of SGK1 on serine 422, this hydrophobic motif in SGK1 has been shown to be the target of mTORC2 (Garcia-Martinez and Alessi, 2008). In support of this, we found reduced band density for p-^*S*422^SGK1 in cortex homogenates in the male mice. However, SGK1 and the ratio of p-^*S*422^SGK1 to total SGK1 band densities were not significantly different. Furthermore, these band densities were not affected by the LS diet in either genotype (Figure 10).

## Discussion

The mechanistic target of rapamycin (mTOR) is a central energy sensor expressed in nearly all cell types (Brown et al., 2018). Using Cre-recombinase-targeted knockout, we deleted mTOR in mice from all cell types expressing Aquaporin 2 (AQP2). This primarily focused the deletion to the principal cells of the distal tubule, including connecting tubule and collecting duct. In male mice, AQP2 is also expressed in several reproductive tissues (Uhlen et al., 2015), including ductus deferens, epididymis, and seminal vesicles, thus only female KO mice were used as breeders to maintain the line. In the principal cells, mTOR, which functions as a kinase, has been demonstrated to be upstream of the ENaC activation by insulin (Pavlov et al., 2013). Therefore, we focused on the regulation of ENaC and electrolyte homeostasis in this set of studies.

In the male KO mice, all three subunits of ENaC were significantly reduced at the protein level, when assessed in cortex homogenates. Furthermore, KO mice excreted significantly reduced amounts of sodium in urine in response to a single injection of benzamil (ENaC) antagonist, suggesting overall ENaC activity was reduced. To test this further, mice were placed on a very-low Na+ (LS) diet for 7-days. KO mice had a retarded ability to reduce sodium losses on the first day of sodium restriction; however, they eventually adapted. Furthermore, KO mice also had attenuated (and somewhat sustained) ability to reduce urinary chloride losses under LS diet. Chloride levels in the Na+ restricted diet were also low at 0.07% of dry weight (vs. 0.67% for the control diet). Thus, mTOR, most likely as a component of mTORC2, facilitates responses in the adaptation of ENaC activity under low-NaCl diet. The retarded ability to reabsorb chloride was likely secondary to reduced depolarization of the apical membrane, as chloride reabsorption is primarily through pendrin in the intercalated cells (which had intact mTOR).

The CD-mTOR KO mice were determined to have modestly lower blood pressure and reduced ENaC subunit expression under normal and low-NaCl diet conditions. The lower blood pressure, we surmise, may have been due to reduced ENaC activity with subsequently reduced plasma volume. We found hematocrit and hemoglobin levels to be higher in the female KO mice, an indication of mild volume contraction. We may speculate that male mice were somewhat more protected from this volume contraction.

In principal cells, mTOR, as a component of the mTORC2 complex, activates the serum and glucocorticoid regulated kinase 1 (SGK1) by phosphorylation in its hydrophobic motif on serine 422 (Lu et al., 2010; Lang and Pearce, 2016). SGK1 is further activated by phosphorylation in the activation loop by PI-3K. Activated SGK1 then phosphorylates NEDD4-2, an E3-ubiquitin ligase, which increases its association with 14-3-3 proteins and reduces its ability to ubiquitinate ENaC subunits. Thus, we hypothesized that KO of mTOR might lead to down-regulation of ENaC abundance by upregulating ubiquitination of ENaC subunits *via* reduced SGK1 activity. In agreement with our hypothesis, using an ELISA approach to pull-down all ubiquitinated proteins from the cortex homogenate, we found significantly increased representation of α-ENaC subunit in this pool in the CD-mTOR KO, vs. the WT, mice. This supports a greater proportion of cellular α-ENaC was ubiquitinated in the CD-mTOR KO mice. We also found reduced levels of phosphorylated SGK1 in cortex homogenates in the KO, further supporting the hypothetical pathway.

However, we did not observe any increase in β- or γ-ENaC ubiquitination, despite these subunits being reduced in total abundance. This might suggest that these two subunits are not as sensitive to this particular recycling regulatory pathway. In fact, γ-ENaC trended toward being reduced with regard to ubiquitination in the KO mice. Subunit differences in their relative ubiquitinated status might also have something to do with the binding of the polyclonal antibodies to their antigenic sites that may be modified by the ubiquitination process. It is important to note that band densities for the major bands for both γ- and β-ENaC show a reduction in density under conditions that have been shown to stimulate ENaC, e.g., low- Na+ diet. Thus, it is possible that these N-terminal region antibodies lose relative sensitivity with the activation of the channel itself. Further studies would be required to flesh this out.

We can compare and contrast our phenotype with those of α-ENaC knockout mice. In one model, the same AQP2-promoter targeting transgene was used to delete α-ENaC (Christensen et al., 2010). A second model knocked out α-ENaC using a doxycycline-inducible PAX8-promoter targeting transgene (Perrier et al., 2016). α-ENaC deletion in these mouse models caused significant sodium wasting, weight loss, and hyperkalemia. Because our model did not completely abolish α-ENaC expression, we can conclude mTOR activity is not essential, but rather is facilitatory of α-ENaC expression. These models did contrast, however, to deletion of α-ENaC using HOXB7 promoter targeting, in which mice showed no impairment in sodium or potassium homeostasis under various challenges (Rubera et al., 2003). While AQP2 and PAX8 are expressed in connecting tubule, as well as, collecting duct, HOXB7 is primarily collecting-duct associated. Taken together these findings highlight the importance of sodium reabsorption in the early distal tubule, i.e., the distal convoluted tubule and connecting tubule.

We also evaluated other major sodium transporters and exchangers of other sites along the renal tubule to evaluate potential compensation. We came up with a few possible compensatory changes that might have alleviated sodium losses in our mice. First NCC, a major aldosterone-regulated protein of the distal convoluted tubule (Wang et al., 2015) was increased about 20% in the KO under both dietary conditions. Surprisingly, we did not find this protein increased by LS diet in these mice, as has been shown in other studies at least with rats (Masilamani et al., 2002). Furthermore, NaPi-2 and NBCe1, sodium reabsorptive proteins of the proximal tubule were marginally increased in the KO, which may have enhanced proximal tubule sodium retention. Since aldosterone levels did not appear elevated in the KO mice (even trended lower), additional studies will be needed to understand these compensations.

Although this study did not set out to capture sex differences in the response to the KO, we did observe a similar abundance level of mTOR in the kidneys of male vs. female mice. However, male, but not female mice seemed to have relatively more mTOR in cortex homogenates, as compared to inner medulla, when equal amounts of protein were loaded, perhaps representing a greater reliance on this pathway in male proximal tubule. Both sexes also demonstrated a reduction in α-ENaC with KO. Female KO mice did show some increase in hematocrit, as well as an increase in urine sodium losses in the basal state, which may have reflected slight volume contraction, that was not as apparent in the male KO. Unfortunately, we did not have the opportunity to conduct all of the original analyses in the female sex that were conducted in the males.

One caveat to consider in interpretation of these data is that deletion of mTOR would result in a reduction in both mTORC1 and mTORC2 activity. In this particular set of studies, we focused primarily on an end-point downstream of mTORC2, i.e., at least as we currently understand it; however, it is possible that some of the changes we found in expression of renal proteins, blood chemistry, or whole-body adaptations to a low-sodium diet were due to changes in mTORC1 signaling and downstream effectors. Additional studies will be needed to fully characterize this aspect of our model system.

## Conclusion

Collecting duct principal-cell-targeted mTOR deletion by Cre-recombinase mediated gene deletion results in a whole-body phenotype of modest salt wasting. At the cellular level, there is a delayed natriferic response to sodium restriction suggesting mTOR activity (along with the much more robust renin-angiotensin-system) facilitates sodium homeostasis under low-NaCl dietary conditions. Thus, dysregulation of this signaling cascade, e.g., during insulin resistance or type 2 diabetes, may hinder whole-body sodium homeostasis.

## Data Availability Statement

The original contributions presented in the study are included in the article/Supplementary Material, further inquiries can be directed to the corresponding author.

## Ethics Statement

The animal study was reviewed and approved by the Georgetown University Institution Animal Care and Use Committee.

## Author Contributions

BC and MF conducted the experiments, performed the analysis, interpreted the data, drafted the manuscript, and proof-read the final manuscript version. SS and AB conducted the experiments, analyzed the data, and finalized the manuscript. AG conducted the experiments, performed the statistical analyses, and proof-read the final manuscript. CE designed the studies, wrote the animal protocols, interpreted the data, graphed the figures, and finalized the manuscript. All authors contributed to the article and approved the submitted version.

## Conflict of Interest

The authors declare that the research was conducted in the absence of any commercial or financial relationships that could be construed as a potential conflict of interest.

## Publisher’s Note

All claims expressed in this article are solely those of the authors and do not necessarily represent those of their affiliated organizations, or those of the publisher, the editors and the reviewers. Any product that may be evaluated in this article, or claim that may be made by its manufacturer, is not guaranteed or endorsed by the publisher.
