# Supplementary material for: Selective Deletion of the Mechanistic Target of Rapamycin From the Renal Collecting Duct Principal Cell in Mice Down-Regulates the Epithelial Sodium Channel
Source: Front Physiol. 2022 Jan 4;12:787521. doi: 10.3389/fphys.2021.787521 (PMC8764147; doi:10.3389/fphys.2021.787521)
Supplement: Supplementary file 1 [file Table_1.DOCX]

| **Table 1S: Weight and Blood Parameters in female CD-PC mTOR KO mice under normal and low-NaCl diets*** | | | | | | | |
| --- | --- | --- | --- | --- | --- | --- | --- |
|  | WT-NS | KO-NS | WT-LS | KO-LS | Two-Way ANOVA (p-values) | | |
|  |  |  |  |  | Genotype | Diet | Interaction |
| Final body weight (g) | 21.5 ± 0.5 | 23.2 ± 1.2 | 21.3 ± 0.5 | 24.1 ± 0.7 | **0.006** | 0.64 | 0.50 |
| Kidney weight (g/25g·bw) | 0.146 ± 0.007 | 0.140 ± 0.004 | 0.150 ± 0.004 | 0.146 ± 0.002 | 0.23 | 0.22 | 0.76 |
| BUN (mg/dL) | 16.7 ± 1.1 | 17.3 ± 1.3 | 15.7 ± 1.7 | 17.3 ± 1.1 | 0.39 | 0.72 | 0.72 |
| Glucose (mg/dL)^†^ | 175 ± 16 | 185 ± 10 | 184 ± 16 | 215 ± 7 | 0.12 | 0.14 | 0.41 |
| Hct (%PCV) | 23.8 ± 1.1^B^ | 26.3 ± 1.3^AB^ | 24.4 ± 2.2^B^ | 31.3 ± 0.8^A^ | **0.0044** | 0.071 | 0.15 |
| Hb (g/dL) | 8.1 ± 0.4^B^ | 9.0 ± 0.4^AB^ | 8.3 ± 0.8^B^ | 10.6 ± 0.3^A^ | **0.0041** | 0.069 | 0.15 |
| pH (log H+) | 7.33 ± 0.02^A^ | 7.33 ± 0.02^AB^ | 7.24 ± 0.02^AB^ | 7.22 ± 0.03^B^ | 0.65 | **0.0016** | 0.77 |
| Beecf (mM) | -1.83 ± 1.82 | 1.00 ± 1.61 | -3.57 ± 2.32 | -0.83 ± 2.63 | 0.24 | 0.32 | 0.86 |
| *mean ± sem (n = 6, 7, 6, and 6 for WT-NS, KO-NS, WT-LS, KO-LS, respectively); ^†^non-fasted; Superscript letters indicate significant differences between groups as determined by Tukey’s multiple comparisons test; “A” (assigned to highest mean) is significantly different from “B”, but not “AB”. | | | | | | | |
