# Supplementary figures and images for: Selective Deletion of the Mechanistic Target of Rapamycin From the Renal Collecting Duct Principal Cell in Mice Down-Regulates the Epithelial Sodium Channel
Source: Front Physiol. 2022 Jan 4;12:787521. doi: 10.3389/fphys.2021.787521 (PMC8764147; doi:10.3389/fphys.2021.787521)

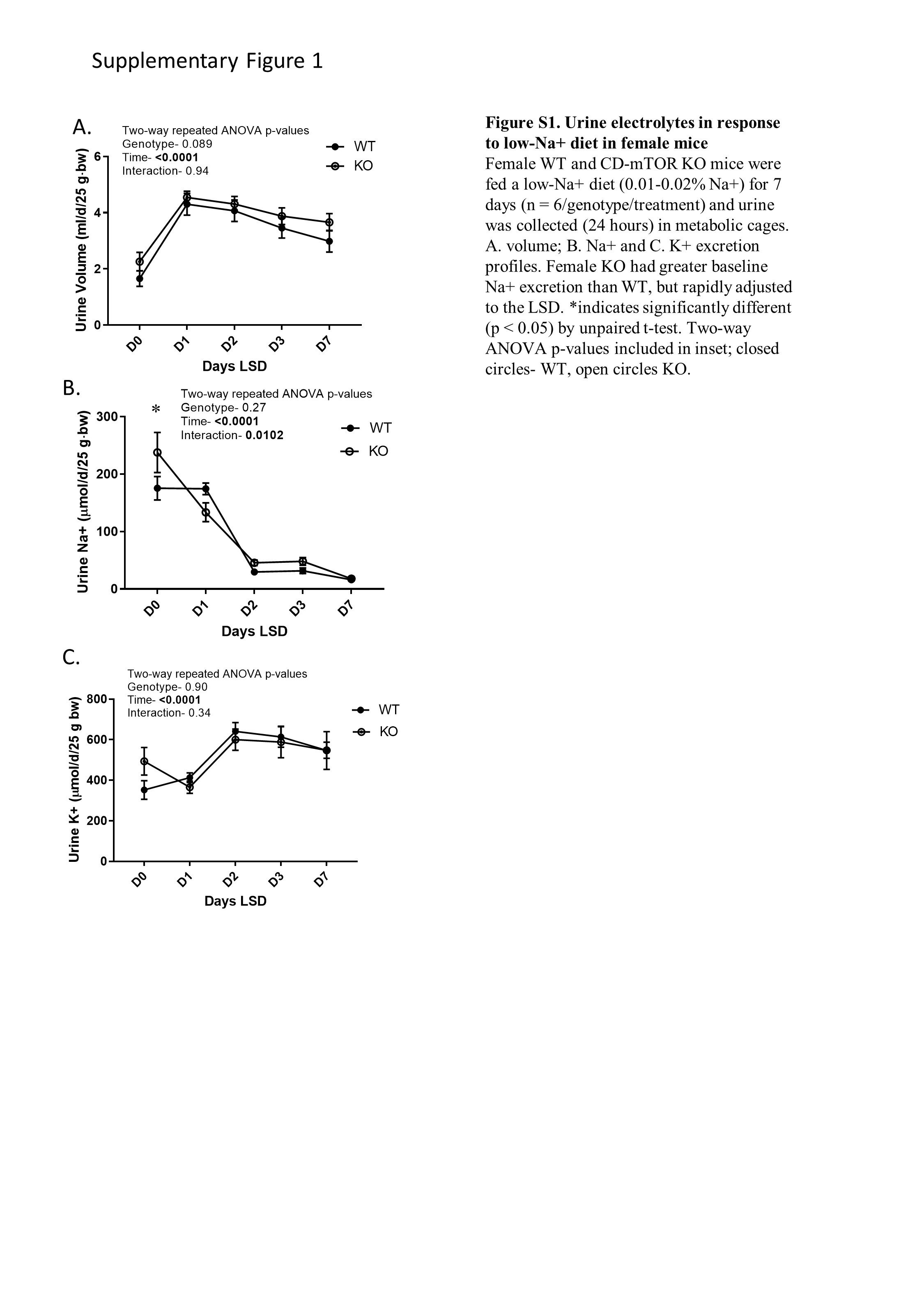

Supplement: Supplementary file 2 [file Image_1.JPEG]

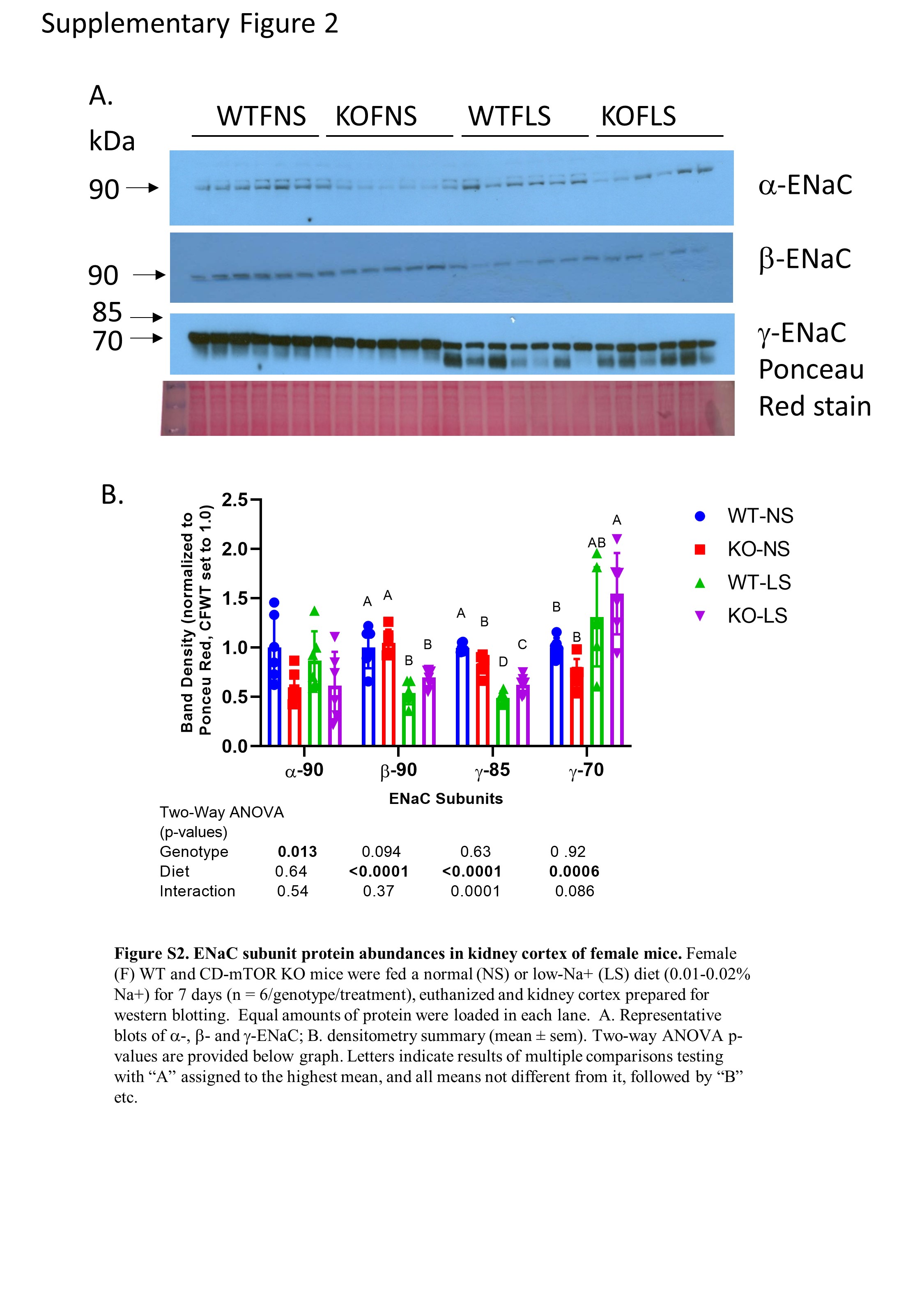

Supplement: Supplementary file 3 [file Image_2.JPEG]
